# Zur nichtkonsensuellen Weiterleitung persönlicher erotischer Fotos an Schulen: Eine Befragung von Schulleitungen in Schleswig-Holstein zu sexuellen Grenzverletzungen mittels digitaler Medien

**DOI:** 10.1007/s00103-021-03436-4

**Published:** 2021-10-18

**Authors:** Arne Dekker, Pia Behrendt, Lea Pregartbauer

**Affiliations:** Institut für Sexualforschung, Sexualmedizin und Forensische Psychiatrie, Universitätsklinikum Hamburg-Eppendorf, Martinistraße 52, 20246 Hamburg, Deutschland

**Keywords:** Sexting, Sexualisierte Gewalt, Digitale Medien, Prävention, Deutschland, Sexting, Sexualized violence, Digital media, Prevention, Germany

## Abstract

**Hintergrund:**

Neben Chancen bringen Internet und digitale Medien für Kinder und Jugendliche auch Risiken mit sich. Ein solches stellen Fälle dar, bei denen persönliche erotische Fotos von Schüler:innen gegen deren Willen weiterverbreitet werden. Für Betroffene können die psychosozialen Konsequenzen gravierend sein.

**Ziel der Arbeit:**

Mit der vorliegenden Arbeit sollen Prävalenz von und Umgang mit der nichteinvernehmlichen Weiterleitung persönlicher erotischer Fotos unter Schüler:innen an Schulen in Schleswig-Holstein erhoben werden.

**Methode:**

Vom 25.04. bis zum 07.06.2019 wurden die Schulleitungen von weiterführenden Schulen mittels eines Onlinekurzfragebogens sowohl zum Vorkommen nichtkonsensueller Fotoweiterleitung an den jeweiligen Schulen befragt als auch zu ergriffenen Maßnahmen, Folgen für die betroffenen Schüler:innen und Konsequenzen für diejenigen, die die Fotos versendet haben. Die Angaben von 74 Schulleitungen konnten in die Datenanalyse aufgenommen werden.

**Ergebnisse:**

An mehr als zwei Dritteln der Schulen wurde den Schulleitungen mindestens ein Fall von nichtkonsensueller Fotoweiterleitung bekannt. Ergriffene Maßnahmen waren zumeist die Information der Eltern aller Beteiligten und ein „angeleiteter Austausch“ zwischen den beteiligten Schüler:innen. Als Folgen für die betroffenen Schüler:innen wurden v. a. sozialer Rückzug, psychisches Leiden, schulische Leistungsprobleme und Erfahrungen mit Cybermobbing/-bullying berichtet. In acht Fällen verließen betroffene Schüler:innen die Schule.

**Diskussion:**

Nichtkonsensuelle Fotoweiterleitung an Schulen ist ein Problem erheblichen Ausmaßes. Zeitgemäße spezifische Präventionsmaßnahmen sind dringend erforderlich.

## Abstract (en)

**Background:**

The Internet and digital media entail both risks and opportunities for children and young people. One risk is posed by cases in which personal erotic photos of schoolchildren are forwarded against their will. For the children and adolescents concerned, the psychosocial consequences can be serious.

**Objective:**

The aim of this study is to investigate the prevalence and consequences of non-consensual forwarding of personal erotic photos among children and adolescents at schools in the German state of Schleswig-Holstein.

**Methods:**

From 25 April to 7 June 2019, principals of secondary schools were surveyed via an online brief questionnaire about both the incidence of non-consensual forwarding of erotic photos at their respective schools, as well as actions taken, consequences for the students depicted, and consequences for those who sent the photos. Information from 74 school administrators was included in the data analysis.

**Results:**

In more than two-thirds of the schools, school principals were aware of at least one case of non-consensual forwarding of personal erotic photos. Measures taken were mostly informing the parents of all involved and a “guided exchange” between the involved students. The consequences for the affected students were reported to be social withdrawal, psychological suffering, school performance problems, and experiences with cyberbullying. In eight cases, affected students left the school.

**Discussion:**

Non-consensual forwarding of personal erotic photos in schools is a problem of considerable magnitude. Up-to-date specific prevention measures are urgently needed.

## Hintergrund

Nicht erst seit einem durch die Coronapandemie ausgelösten Digitalisierungsschub an Schulen nutzen die allermeisten Kinder und Jugendlichen mit großer Selbstverständlichkeit digitale Medien. Soziale Netzwerke stellen für sie – ebenso wie für viele Erwachsene – zentrale Kommunikationsräume dar. Dies ist mit zahlreichen Chancen, aber auch mit Risiken verbunden. So spricht vieles dafür, dass sexuelle Grenzverletzungen mittels digitaler Medien gegenüber Kindern und Jugendlichen in jüngster Zeit auch angesichts der COVID-19-bedingten vermehrten Isolation noch einmal zugenommen haben [1]. Das Spektrum digitaler Grenzverletzungen ist enorm: Es reicht von ungewollten Kontaktaufnahmen und Flirts online über die gezielte digitale Vorbereitung von sexualisierter Gewalt in realen Räumen (Online-Grooming) bis hin zu Grenzverletzungen und Gewalt mittels bildlicher Darstellungen, also z. B. der Produktion, Verbreitung und Nutzung von Missbrauchsdarstellungen (für eine ausführliche Darstellung s. u. a. [2]). Der vorliegende Beitrag widmet sich nur einem sehr kleinen Ausschnitt dieses Spektrums, der jedoch an Schulen von besonderer Bedeutung ist: der nichteinvernehmlichen Weiterleitung persönlicher erotischer Fotos unter Schüler:innen.

Als „Sexting“ wird heute meist eine Praxis bezeichnet, bei der mittels Smartphones persönliche erotische Fotos aufgenommen und willentlich an andere Personen versandt werden. Dabei kann der Inhalt der Fotos variieren, von Badehosen- oder Bikinifotos über Nacktfotos bis hin zu sexuellen Darstellungen, mit oder ohne Zeigen des Gesichts usf. Obwohl Sexting für sich genommen nicht problematisch sein muss, häufig konsensuell stattfindet (z. B. zwischen Beziehungspartner:innen) und von vielen Erwachsenen regelmäßig praktiziert wird (vgl. [3]; für eine systematische Übersicht s. [4]), wird Sexting unter Jugendlichen sowohl im wissenschaftlichen als auch im Präventionsdiskurs oft als mediales Problemverhalten interpretiert [5]. Tatsächlich kann Sexting zum Ausgangspunkt einer gravierenden sexuellen Grenzverletzung mit teils erheblichen psychosozialen Folgen für die Betroffenen werden, wenn die versandten Fotos von Empfänger:innen gegen den Willen der abgebildeten Personen an Dritte weitergeleitet werden, beispielsweise im Freundeskreis oder im Klassenverband. Die Charakterisierung von Sexting als Risiko- oder Problemverhalten per se ist jedoch auch fragwürdig, weil die Verantwortung für das Geschehen dadurch den abgebildeten Betroffenen zugeschrieben wird, während die nichtkonsensuelle und z. T. massenweise Weiterleitung der Fotos beispielsweise unter Mitschüler:innen bagatellisiert oder ausgeblendet wird (s. u. a. [2, 5, 6]). Dass ausgerechnet beim gleichzeitigen Auftreten zweier an Schulen häufig als problematisch wahrgenommener Phänomene – nämlich Sexualität und Nutzung digitaler Medien – die Frage nach dem Konsens aus dem Blick gerät, haben wir an anderer Stelle als „doppelten Verdeckungszusammenhang“ von Sexualität und digitalen Medien beschrieben [7]. Für die von nichtkonsensueller Bildweiterleitung betroffenen Schüler:innen führt diese Ausblendung von Konsensualität nicht selten zu Erfahrungen des *Victim Blaming *(dt. Täter-Opfer-Umkehr). Während einige Daten zum Sexting-Verhalten Jugendlicher sowohl national als auch international vorliegen, existieren kaum Daten zur nichtkonsensuellen Weiterleitung von Sexting-Fotos. In einer Onlineerhebung [8] unter 14- bis 17-jährigen Jugendlichen gaben 28 % der 254 Befragten an, schon ein- oder mehrmals ein erotisches Foto oder Video an andere Jugendliche verschickt zu haben, auf dem sie selbst nackt oder halbnackt zu sehen waren. Von diesen aktiven Sexter:innen berichten 10 %, „dass ein von ihnen verschicktes Foto/Video schon einmal ohne ihr Einverständnis an andere weitergeleitet oder veröffentlicht wurde“, was einer Gesamtprävalenz von knapp 3 % entspricht [9]. Auch im Rahmen der Speak-Studie, für die in Hessen 2719 Schüler:innen an allgemeinbildenden Schulen befragt wurden, wurde die nichtkonsensuelle Bildweiterleitung thematisiert – hier erklärten 0,9 % der Jungen und 2 % der Mädchen, jemand habe „gegen meinen Willen intime Fotos oder Filme von mir ins Internet gestellt“ [10]. Zusatzerhebungen der Speak-Studie an Förderschulen und an Berufsschulen kommen zu ähnlichen Prävalenzraten [11, 12].

Auf Basis der begrenzten Datenlage können wir also sagen, dass eine Minderheit Jugendlicher über Erfahrungen mit Sexting verfügt und hiervon wiederum einer Minderheit die nichtkonsensuelle Weiterleitung ihrer Bilder widerfährt. Angesichts der u. U. enormen Folgen für die Abgebildeten, die bis hin zum Suizid der Betroffenen reichen können [13], und auch angesichts der Bedeutung vermeintlicher Einzelfälle für die Institution Schule ist es jedoch unerlässlich, sich mit dieser spezifischen Form einer sexuellen Grenzverletzung auseinanderzusetzen.

Mit dem vorliegenden Beitrag möchten wir die Frage beantworten, welches Ausmaß das Problem der nichtkonsensuellen Weiterleitung persönlicher erotischer Fotos an Schulen in Schleswig-Holstein hat und wie mit diesen Fällen umgegangen wird.

## Methode

Die vorliegende Untersuchung war als Onlinebefragung der Schulleitungen aller weiterführenden Schulen im Bundesland Schleswig-Holstein angelegt. Die Rekrutierung erfolgte sowohl postalisch als auch per E‑Mail. Die Befragung war von April 2019 bis Juni 2019 aktiv.

### Das Erhebungsinstrument

Das Erhebungsinstrument war ein Kurzfragebogen, mit dem in wenigen Schritten allgemeine Bedingungen und Regeln der Mediennutzung an der Schule (5 Items bzw. Itemkomplexe), Vorkommen und Umgang mit nichteinvernehmlicher Fotoweiterleitung, Pornografiekonsum an der Schule (4 Items bzw. Itemkomplexe) sowie Präventionsmaßnahmen und sexualbezogene Lerninhalte (2 Items) erfasst wurden. Zudem wurden einige soziodemografische Merkmale erfragt. Die Fragen wurden mittels des Open Source Software Tools LimeSurvey (LimeSurvey GmbH 2003, Hamburg, Deutschland) programmiert und von den Schulleitungen online ausgefüllt.

Für die vorliegende Untersuchung wurden folgende Merkmale ausgewertet:

#### Schulform*.*

„Welcher Schulform ist Ihre Schule zugeordnet?“, mit den Antwortmöglichkeiten „Gemeinschaftsschule“ und „Gymnasium“.

#### Prävalenz nichtkonsensueller Weiterleitung persönlicher erotischer Fotos*.*

„Sind Ihnen Fälle (auch Verdachtsfälle) bekannt, in denen persönliches erotisches Bildmaterial gegen den Willen der abgebildeten Person weitergeleitet wurde?“, mit den Antwortmöglichkeiten „ja, das ist schon einmal vorgekommen“, „ja, das ist mehr als einmal vorgekommen“ und „nein“.

#### Ergriffene Maßnahmen*.*

„Welche der folgenden Maßnahmen hat die Schule ergriffen? Mehrere Antworten sind möglich“, mit den Antwortmöglichkeiten „mir sind keine konkreten Maßnahmen bekannt“, „externe Fachberatungsstelle wurde hinzugezogen“, „Schulbehörde/Ministerium wurde hinzugezogen“, „es erfolgte offene Kommunikation im Klassen‑/Schulverband“, „es erfolgte ein angeleiteter persönlicher Austausch zwischen den beteiligten Schüler:innen“, „es erfolgte ein Austausch im Kollegium“, „die Eltern der Beteiligten wurden informiert“, „Regeln der Mediennutzung wurden verschärft“, „keine Angabe“ und „sonstiges“ mit Freitextfeld für offene Erörterung.

#### Konsequenzen für betroffene Schüler:innen*.*

„Welche Konsequenzen hatte der Fall für die Person, die auf dem Bildmaterial abgebildet war? Mehrere Antworten sind möglich“, mit den Antwortmöglichkeiten „mir sind keine Konsequenzen bekannt“, „Cyberbullying/-mobbing“, „psychisches Leiden“, „sozialer Rückzug“, „schulische Leistungsprobleme“, „Schulwechsel“, „keine Angabe“ und „sonstiges“ mit Freitextfeld für offene Erörterung.

#### Konsequenzen für die grenzverletzenden Schüler:innen*.*

„Gab es Konsequenzen für diejenigen Schüler:innen, die das Bildmaterial gegen den Willen der abgebildeten Person weitergeleitet haben? Mehrere Antworten sind möglich“, mit den Antwortmöglichkeiten „mir sind keine Konsequenzen bekannt“, „Schulwechsel“, „keine Angabe“ und „sonstiges“ mit Freitextfeld für offene Erörterung.

#### Einbezug der Schulleitungen*.*

„Wie hoch schätzen Sie die Wahrscheinlichkeit ein, dass Sie als Schulleitung von derartigen Fällen in Kenntnis gesetzt werden bzw. worden wären?“ Antwort mittels Schieberegler auf 10-stufiger Likert-Skala von 0 („überhaupt nicht wahrscheinlich“) bis 10 („sehr wahrscheinlich“).

#### Bedarf an Aus- und Fortbildung*.*

„Wie hoch ist nach Ihrer Meinung der Bedarf an Aus- und Fortbildungsmöglichkeiten für Lehrkräfte zum Thema ‚Sexuelle Grenzverletzungen mittels digitaler Medien‘?“ Antwort mittels Schieberegler auf 10-stufiger Likert-Skala von 0 („überhaupt nicht hoch“) bis 10 („sehr hoch“).

#### Offene Frage nach sexualbezogener Mediennutzung durch Schüler:innen*.*

„Wenn Sie nun noch einmal allgemein an sexualbezogene Mediennutzung durch Schüler:innen denken – möchten Sie noch etwas zu dem Thema ergänzen?“

### Datenerhebung und Stichprobe

Die Rekrutierung der Schulleitungen erfolgte vom 25.04. bis zum 07.06.2019 in vier Schritten. Zunächst wurde ein postalisches Anschreiben verschickt, mit dem über die Studie informiert und um Mitarbeit gebeten wurde. Das Anschreiben enthielt sowohl einen Link als auch einen QR-Code, die direkt zum Onlinefragebogen führten. Nach zwei und nach vier Wochen wurden Erinnerungsmails verschickt, mit denen erneut um eine Teilnahme gebeten wurde. Abschließend wurde – wiederum per Post – ein Dankesbrief versandt.

Insgesamt wurde die Grundgesamtheit (*N*) aller 281 Schulleiterinnen und Schulleiter der Gymnasien und Gemeinschaftsschulen in Schleswig-Holstein angeschrieben. 104 Schulleitungen riefen den Fragebogen auf, was einer Kooperationsquote von 37,0 % entspricht. Eine Stichprobe (*n*) von 74 Schulleitungen bearbeitete den Fragebogen vollständig und konnte in die Datenanalyse aufgenommen werden. Damit liegt die Teilnahmequote bei 26,3 %, was kein hoher, jedoch ein auch für aufwendige wissenschaftliche Befragungen gegenwärtig typischer Wert ist.

Die Befragung fand im Rahmen des vom Bundesministerium für Bildung und Forschung (BMBF) geförderten Verbundvorhabens „Safer Sexting. Über sexuelle Grenzverletzungen mittels digitaler Medien an Schulen“ statt, das vom Institut für Sexualforschung, Sexualmedizin und Forensische Psychiatrie des Universitätsklinikums Hamburg-Eppendorf in Kooperation mit der Abteilung Schulpädagogik der Universität Flensburg durchgeführt wurde. Ein positives Votum der Ethikkommission der Deutschen Gesellschaft für Erziehungswissenschaft (DGfE) liegt vor. Die Erhebung wurde durch das Ministerium für Bildung, Wissenschaft und Kultur in Schleswig-Holstein genehmigt.

### Auswertung

Die quantitativen Daten wurden mithilfe der Software SPSS Statistics for Macintosh (Version 27.0, IBM Corp. 2020, Armonk, NY, USA) deskriptiv ausgewertet. Dargestellt werden die absoluten und relativen Häufigkeiten bzw. die Mittelwerte samt zugehöriger 95 %-Konfidenzintervalle (95 %-KI). Die KI wurden unter Verwendung des Moduls Bootstrapping bei einfacher Ziehung von 1000 Stichproben berechnet.

Die Antworten auf die offene Frage wurden thematisch codiert und zusammenfassend dargestellt.

## Ergebnisse

### Vorkommen nichtkonsensueller Weiterleitung persönlicher erotischer Fotos an Schulen

**Figure 1:**
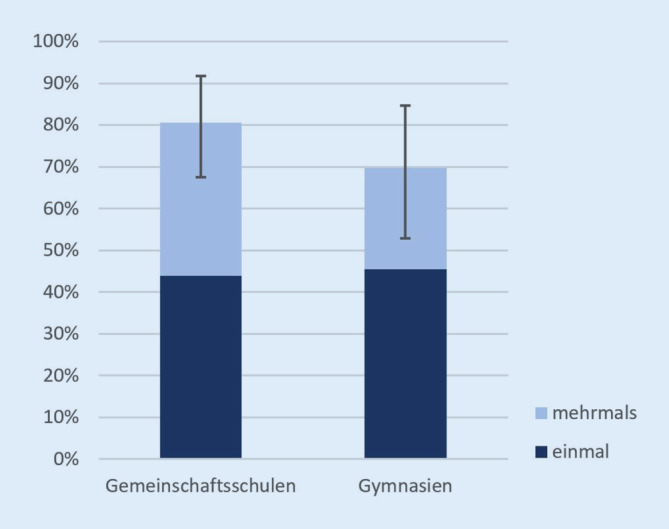

An mehr als zwei Dritteln der Schulen (*n* = 56) berichten die befragten Schulleitungen davon, dass ihnen an ihrer Schule Fälle bekannt geworden seien, bei denen persönliches erotisches Bildmaterial gegen den Willen der abgebildeten Person weitergeleitet worden sei. An einem beachtlichen Teil der Schulen (Gemeinschaftsschulen: 36,6 % [95 %-KI 21,4–52,5 %]; Gymnasien: 24,2 % [95 %-KI 10,3–39,4 %]) ist dies sogar mehr als einmal passiert (Abb. 1).

Die Häufigkeit des Vorkommens nichtkonsensueller Weiterleitung unterscheidet sich zwischen den Schulformen nicht signifikant. Da dies für die weiteren Ergebnisse ebenfalls gilt, verzichten wir im Folgenden auf eine Differenzierung nach Schulform.

### Umgang mit nichtkonsensueller Weiterleitung persönlicher erotischer Fotos an Schulen

Für die 56 Schulen, an denen den Schulleitungen eine nichtkonsensuelle Fotoweiterleitung bekannt geworden ist, zeigt Tab. 1 verschiedene Aspekte des Umgangs damit. Unter den Maßnahmen, die von den Schulen ergriffen wurden, stehen die Information der Eltern aller Beteiligten sowie ein „angeleiteter Austausch“ der beteiligten Schüler:innen an den ersten beiden Stellen. In etwas mehr als der Hälfte der Fälle erfolgte ein Austausch im Kollegium, wurden externe Fachberatungsstellen hinzugezogen und fand ein „offener Austausch“ im Klassen- oder Schulverband über das Thema statt. Deutlich seltener wurden Ministerium bzw. Schulbehörde hinzugezogen oder die Regeln zur Mediennutzung an der Schule geändert. Darüber hinaus berichteten sieben Schulleitungen von „sonstigen“ Maßnahmen und beschrieben in einem Freitextfeld u. a. Strafanzeigen bzw. das Hinzuziehen der Polizei (4 Schulen), Einschaltung der Schulsozialarbeit (2 Schulen), Durchführung von Präventionsseminaren u. a. mit Medienscouts (2 Schulen) sowie informierende Elternabende (eine Schule).

**Table 1:** 

	Anzahl Antworten	Anteil in %	95 %-KI^a^
*Welche Maßnahmen wurden von der Schule ergriffen?*			
Eltern der Beteiligten wurden informiert	50	89,3	80,4–96,4
Angeleiteter Austausch zw. beteiligten Schüler:innen	41	73,2	62,5–83,9
Austausch im Kollegium	32	57,1	42,9–69,9
Externe Fachberatungsstelle wurde hinzugezogen	31	55,4	42,9–67,9
Offene Kommunikation im Klassen‑/Schulverband	31	55,4	42,9–67,9
Ministerium/Schulbehörde wurde hinzugezogen	8	14,3	5,4–23,2
Regeln der Mediennutzung an der Schule wurden verschärft	4	7,1	1,8–14,3
Sonstiges	7	12,8	3,6–21,4
Keine konkreten Maßnahmen bekannt	1	1,8	0,0–5,4
*Welche Konsequenzen hatte der Fall für den:die abgebildete:n Schüler:in?*			
Sozialer Rückzug	24	42,9	30,4–57,1
Psychisches Leiden	23	41,1	28,6–53,6
Schulische Leistungsprobleme	15	26,8	16,1–39,3
Cyberbullying/Cybermobbing	13	23,2	12,5–33,9
Schulwechsel	8	14,3	5,4–23,2
Sonstiges	5	8,9	1,8–17,9
Keine Konsequenzen bekannt	12	21,4	10,7–33,9
Keine Angabe^b^	6	10,7	3,6–19,6
*Gab es Konsequenzen für Schüler:innen, die Bilder weitergeleitet haben?*			
Schulwechsel	4	7,1	1,8–14,3
Sonstiges	38	67,9	55,4–80,3
Keine Konsequenzen bekannt	4	7,1	1,8-14,3
Keine Angabe^b^	10	17,9	8,9–28,6

^a^Berechnung des Konfidenzintervalls basierend auf 1000 einfachen Bootstrap-Stichproben

^b^Ausschlussantwort

Explizit nach den Folgen für die betroffenen Schüler:innen, deren Bilder weitergeleitet wurden, gefragt, berichteten die meisten Schulleitungen von sozialem Rückzug und psychischem Leiden, darüber hinaus in rund einem Viertel der Fälle auch von schulischen Leitungsproblemen und Erfahrungen mit Cybermobbing/-bullying. An immerhin 8 Schulen haben die betroffenen Schüler:innen die Schule verlassen, was im Kontrast zu der Tatsache steht, dass in nur 4 Fällen jene Schüler:innen die Schule gewechselt haben, die Bilder gegen den Willen der Abgebildeten weitergeleitet haben (s. unten). 5 Befragte machten zudem „sonstige“ Angaben, die jedoch lediglich Erläuterungen der Fälle und keine zusätzlichen Konsequenzen für die Betroffenen enthielten.

Nach den Konsequenzen für jene Schüler:innen gefragt, die die Grenzen anderer durch die nichteinvernehmliche Weiterleitung persönlicher erotischer Fotos verletzt haben, machte ein knappes Fünftel der befragten Schulleitungen keine Angaben. Wie oben bereits erwähnt, berichteten 4 Schulleitungen über einen Schulwechsel der grenzverletzenden Schüler:innen. Eine besondere Rolle spielte bei dieser Frage zudem die Angabe „Sonstiges“: 38 Schulleitungen (67 %) gaben dies an und immerhin 23 berichteten über verschiedene Maßnahmen nach § 25 SchulG (schleswig-holsteinisches Schulgesetz) für die grenzverletzenden Schüler:innen, darunter u. a. Missbilligung, schriftlicher Verweis, Klassenwechsel und zeitweiser Ausschluss vom Unterricht. In 7 Fällen wurde die Polizei hinzugezogen, teilweise wurde Strafanzeige erstattet. Berichtet wurde außerdem über Elterngespräche (2 Schulen) und normenverdeutlichende Gespräche mit den Schüler:innen (2 Schulen).

### Weitere Einschätzungen der Schulleitung

Die befragten Schulleitungen schätzten die Wahrscheinlichkeit, über eine nichtkonsensuelle Bildweiterleitung informiert zu werden, auf einer 10-stufigen Likert-Skala im Durchschnitt als eher hoch ein – ein Befund, der durch das bereits dargestellte häufige Vorkommen an Schulen gestützt wird (Tab. 2). Nach dem Aus- und Fortbildungsbedarf zum hier behandelten Themenkomplex befragt, lagen die Schulleitungen in der Bewertung im Schnitt nur knapp über einem mittleren Wert.

**Table 2:** 

	M (sd)^a^	95 %-KI^b^
*Wahrscheinlichkeit, dass Sie als Schulleitung in Kenntnis gesetzt werden* (0 = überhaupt nicht wahrscheinlich bis 10 = sehr wahrscheinlich)	7,1 (2,4)	6,5–7,6
*Bedarf an Aus- und Fortbildungsmöglichkeiten für Lehrkräfte zum Thema* (0 = überhaupt nicht hoch bis 10 = sehr hoch)	6,0 (2,3)	5,4–6,5

^a^Arithmetisches Mittel (Standardabweichung)

^b^Berechnung des Konfidenzintervalls basierend auf 1000 einfachen Bootstrap-Stichproben

Neben diesen standardisierten Einschätzungen wurden die Schulleitungen zudem mit einer offenen Frage um ergänzende Einschätzungen zur sexualbezogenen Mediennutzung durch Schüler:innen gebeten, wovon 17 Befragte Gebrauch machten. Tab. 3 zeigt die zusammengefassten Ergebnisse der thematischen Codierung der Antworten. Auch hier sahen die Schulleitungen die Einflussmöglichkeiten von Schule als gering an, sahen die zentrale Verantwortung beim Elternhaus und beschrieben vorhandene Präventionsmaßnahmen als ausreichend. Lediglich zwei Schulleitungen erkannten einen zusätzlichen Aus- und Fortbildungsbedarf für Lehrpersonen.

**Table 3:** 

Kategorie	Antwortbeispiel	Anzahl Antworten
Schulen haben keine Kontrollmöglichkeiten	Für Schule ist dies ein schwieriger und heikler Bereich, da Schulleitung datenschutzrechtlich keine Handhabe hat. Lehrkräfte erfahren von solchen Dingen, die aber in der Freizeit/am Nachmittag/am Wochenende der Schüler*innen geschehen. Somit fällt dies außerhalb unserer Eingriffsmöglichkeiten, obwohl es durchaus Relevanz für den Unterrichtsvormittag hat. Nur hier kommen die betreffenden Schüler*innen zusammen und sehen sich Face-to-Face	6
Die Verantwortung liegt (auch) bei den Eltern	In den Elternhäusern ist die Nutzung von Smartphones z. T. kaum bis gar nicht reglementiert. In der Schule ist ständig ein gepflegter Contentfilter aktiv. So etwas ist für die Schüler zu Hause oft nicht vorhanden. Die Nutzungszeiten werden zusätzlich oft kaum überwacht. Das hat auch Gründe im fehlenden Know-how	5
Weitere Fortbildungsbedarfe für Lehrer:innen	Mediennutzung jeglicher Art ist omnipräsent. Schule hat die Aufgabe, diesem Thema pädagogisch zu begegnen. Mit Verboten ist nichts gewonnen. Überdies überwiegt der Nutzen der modernen digitalen Medien und Geräte und ist unverzichtbar. Aufgabe der Schule ist es, das Thema medienpädagogisch anzunehmen. Problematisch wirkt sich die Unkenntnis und Nutzungsabneigung (unterentwickelte Medienkompetenz) so mancher Lehrpersonen aus	2
Präventionsmaßnahmen sind bereits implementiert	Themen sind entsprechend der Altersstufen in Unterrichtseinheiten zur Gewaltprävention eingebunden	2
Schüler:innen überschätzen ihre eigene Medienkompetenz	Viele Schüler*innen sind sehr neugierig und teilen Filme, ohne zu verstehen. Sie überschätzen ihr eigenes Wissen dadurch sehr	2

## Diskussion

Frühere Ergebnisse [8–12] zeigen für die nichtkonsensuelle Weiterleitung persönlicher erotischer Fotos unter Schüler:innen Viktimisierungsraten zwischen 0,9 % und knapp 3 %, wobei die Folgen für einzelne Betroffene z. T. gravierend sein können. Die Ergebnisse der vorliegenden Untersuchung zeigen, wie gravierend das Problem aus der Sicht der Institution Schule ist: An mehr als zwei Dritteln der weiterführenden Schulen in Schleswig-Holstein sind den befragten Schulleitungen Fälle dieser Form sexueller Grenzverletzungen bekannt geworden, an vielen davon mehrfach. Die Schulleitungen schätzen die z. T. schweren Konsequenzen für die betroffenen Schüler:innen vermutlich realistisch ein und nennen auch eine Reihe von ergriffenen Maßnahmen (insb. Austausch mit Eltern, zwischen beteiligten Schüler:innen, im Kollegium, im Schul- oder Klassenverband, mit Fachberatungsstellen sowie Ordnungsmaßnahmen gegenüber den sexuell grenzverletzenden Schüler:innen). Aber obwohl eine Bewertung der Maßnahmen auf Grundlage unserer begrenzten Daten auf Einzelfallebene schwierig ist, bleibt der Eindruck erheblicher Hilflosigkeit. So haben mehr betroffene als grenzverletzende Schüler:innen nach dem Vorfall die Schule verlassen und in mehr als einem Viertel der Fälle sind den Schulleitungen entweder keine Konsequenzen für die grenzverletzenden Schüler:innen bekannt oder sie machen keine Angaben zu diesen Konsequenzen.

Das zentrale Ergebnis der vorliegenden Untersuchung bleibt aus Sicht der Autor:innen aber das regelmäßige Vorkommen nichtkonsensueller Weiterleitung persönlicher erotischer Fotos an Schulen. Schulleitungen weiterführender Schulen, so scheint es, müssen früher oder später mit dem Bekanntwerden einer nichtkonsensuellen Bildweiterleitung an ihrer Schule rechnen. Die Hoffnung, die eigene Schule möge verschont bleiben, ist also gewiss kein guter Ratgeber. Anders als bei anderen Dimensionen von Sexualität, so zumindest der Eindruck, gelingt es an vielen Schulen nicht, Konsens als zentrale Voraussetzung für sexualbezogene Mediennutzung zu etablieren. In ihren offenen Antworten sehen mehrere Schulleitungen die Schulen mit der Kontrolle überfordert und sehen die Eltern in der Pflicht. Gleichzeitig – und durchaus überraschend – schätzen sie den Fortbildungsbedarf für Lehrer:innen als nicht besonders hoch ein. Dieses Gefühl der mangelnden Zuständigkeit spiegelt sich bisweilen auch in qualitativen Daten, die wir im Rahmen von Gruppendiskussionen mit Lehrer:innen erhoben haben (s. u. a. [14]). Insgesamt bleibt der Eindruck, dass die nichtkonsensuelle Fotoweiterleitung an Schulen ein sexualbezogenes Gesundheitsproblem erheblichen Ausmaßes darstellt, das neben allgemeinen Medienkompetenztrainings spezifische Präventionsmaßnahmen erforderlich macht.

### Limitationen

Für die vorliegende Studie haben wir Schulleitungen befragt, ob ihnen an ihrer Schule Fälle bekannt sind, in denen persönliches erotisches Bildmaterial gegen den Willen der abgebildeten Person weitergeleitet wurde. Voraussetzung für eine Nennung ist also, dass die Schulleitung vom jeweiligen Vorfall erfahren hat. Es ist davon auszugehen, dass manche Fälle nichtkonsensueller Bildweiterleitung zwar Lehrer:innen bekannt werden, nicht aber den Schulleitungen. Eine Befragung von Lehrer:innen ließ sich aber weder forschungspragmatisch um- noch behördlich durchsetzen. Wir gehen dennoch davon aus, dass die hier vorgestellten Prävalenzraten eine gute Schätzung jener Fälle bieten, in denen die nichtkonsensuelle Weiterleitung zu einem größeren Problem an der Schule geworden ist.

Eine weitere Einschränkung der Methode besteht darin, dass über die Kurzbefragung der Schulleitungen weder die Inhalte des jeweils weitergeleiteten Bildmaterials noch die konkreten Abläufe der Vorfälle erfasst werden konnten. Künftige, v. a. qualitative Studien könnten hier einen wichtigen Beitrag leisten.

Als Limitation muss schließlich genannt werden, dass die Teilnahmequote mit 26,3 % zwar nicht unüblich, dennoch aber als relativ niedrig einzuschätzen ist. Obwohl wir eine Vollerhebung der Schulleitungen weiterführender Schulen in Schleswig-Holstein angestrebt haben, können wir nicht von einer Repräsentativität der Daten für Schulen in Schleswig-Holstein ausgehen. Dennoch zeichnet die Datenauswertung ein ebenso wichtiges wie unerfreuliches Bild von einem zentralen Problem sexualbezogener Mediennutzung an Schulen.
